# Supplementary material for: How We Manage Bone Marrow Edema—An Interdisciplinary Approach
Source: J Clin Med. 2020 Feb 18;9(2):551. doi: 10.3390/jcm9020551 (PMC7074543; doi:10.3390/jcm9020551)
Supplement: Supplementary file 1 [file jcm-09-00551-s001.pdf]

Table.S1: Literature overview of molecular, immunological and histopathological findings in BME..

Reference	Year	Study Design	disease	location	*in vivo/ in vitro*	species	gene/ molecule	increased/ decreased	treatment	comment
1	2003	Case report	Subchondral insufficiency fracture (SIF)	Radiological, Histology	*in vivo*	Homo sapiens	n.a.	n.a.	n.a.	dense irregular, discontinued trabeculae
2	2017	Animal model	Bone marrow lesion	Radiological, Histology	*in vivo*	Oryctolagus cuniculus	n.a.	n.a.	n.a.	Biomechanical BME induction and MRI
3	2018	Cohort Study	Spondyloarthropathy	Serum	*in vivo*	Homo sapiens	GDF15	increased	n.a.	
4	2019	Interventional Study	Spondyloarthropathy	Serum	*in vivo*	Homo sapiens	DKK1	decreased	TNF antibody	
5	2011	Genetic animal model	Spondyloarthropathy	Radiological, Histology	*in vivo*	Mus musculus	n.a.	n.a.	n.a.	Biomechanical BME induction and MRI, TNF is essential for the induction and maintenance of BME, increased vascularity and cellularity
6	2010	Genetic animal model	Spondyloarthropathy	Radiological, Histology	*in vivo*	Mus musculus	n.a.	n.a.	n.a.	Biomechanical BME induction and MRI, no significant increase of BME in TNFtg mice, but sig. Increase of osteoclasts and vascularity
7	2013	Cohort Study	Axial psoriatic arthritis, axial spondyloarthritis, ankylosing spondylitis	Serum	*in vivo*	Homo sapiens	HLA-B27	increased	n.a.	HLA-B27 positive correlation to BME
8	2006	Case series	Ankylosing spondylitis	Histology	*in vivo*	Homo sapiens	CD3	increased	n.a.	T cells
							CD4	increased		T cells
							CD8	increased		T cells
							CD20	increased		B cells
							CD34	increased		vessels
							HLA-B27	increased		
9	2012	Cohort Study	Ankylosing spondylitis	Serum	*in vivo*	Homo sapiens	MMP3	increased	n.a.	
10	2017	Cohort Study	Undifferentiated arthritis	Serum, Synovia	*in vivo*	Homo sapiens	Galectin-3	increased	n.a.	
							PIIANP	decreased		
11	2016	Case series	Rheumatoid arthritis	Radiologic, Serum	*in vivo*	homo sapiens	anti-citrullinated protein antibodies (ACPA)	increased	n.a.	
							rheumatoid factor (RF)	increased		
							anti-carbamylated protein (anti-CarP)	increased		
12	2018	Genetic animal model	Rheumatoid arthritis	Histology	*in vivo*	Mus musculus	CD3	increased	n.a.	Tyro3/Axl/Mertk-deficient mice, T cells
							CD19	increased		B cells
							ADGRE1	increased		macrophages
13	2007	Genetic animal model, Human sample	Osteoarthritis	Radiological	*in vivo*	Homo sapiens<br>Cavia porcellus	n.a.	n.a.	n.a.	Reduced tibia perfusion

14	2005	Animal model	Osteoarthritis	Radiological	*in vivo*	Canis lupus familiaris	n.a.	n.a.	n.a.	
15	2008	Case series	Osteoarthritis	Radiological, Histology	*in vivo*	Homo sapiens	n.a.	n.a.	n.a.	abnormal swollen adipocytes, healing micro-fractures, cyst formation, thickened subchondral plate/subchondral sclerosis
16	2019	Cohort study	Osteoarthritis	Radiological, Serum	*in vivo*	Homo sapiens	RETN	increased	n.a.	
17	2019	Cohort study	Osteoarthritis	Radiological, Histology	*in vivo*	Homo sapiens	n.a.	n.a.	n.a.	Bone morphology changes in the anterior-medial (AM) region
18	2016	Cohort study	Osteoarthritis	Radiological, Histology	*in vivo*	Homo sapiens	vWF<br>CD31	increased<br>increased	n.a.	increased bone turnover, increased vascularity, reduced fat
19	2019	Animal model	Osteoarthritis	Radiological, Synovia, Histology, *In vitro* experiments	*in vivo*	Mus musculus	CD11b<br>TRAP<br>CD254	increased<br>increased<br>increased	n.a.	collagen-AB induced OA, decrease of trabecula, increase of B/T cells
20	2019	Interventional study	Osteoarthritis	Radiological	*in vivo*	Homo sapiens	n.a.	n.a.	vitamine D	no effect
21	2014	Interventional study	Osteoarthritis	Radiological, Urine	*in vivo*	Homo sapiens	n.a.	n.a.	glucosamine	no treatment benefit
22	2004	Case series	Chondroblastoma	Radiological, Histology	*in vivo*	Homo sapiens	Cyclooxygenase-2	increased	n.a.	
23	2008	Animal model	Avascular necrosis	Radiological, Histology	*in vivo*	Oryctolagus cuniculus	VEGF	decreased	n.a.	corticosteroid injection, reduced bone
24	2008	Case series	Osteonecrosis	Serum	*in vivo*	Homo sapiens	Kininogen variant 1<br>Complement factor C3 precursor<br>Complement factor H<br>Apolipoprotein A-IV precursor<br>Antithrombin III chain B<br>Gelsolin isoform α precursor	increased<br>increased<br>increased<br>decreased<br>decreased<br>decreased	n.a.	
25	2018	Cohort study	Avascular necrosis	Serum	*in vivo*	Homo sapiens	TNF<br>IL6	decreased<br>decreased	hyperbaric oxygen	
26	2009	Case Report	Hypophosphatasia	Radiological, Histology	*in vivo*	Homo sapiens	ALPL	SNP	n.a.	bone necrosis, marrow fibrosis
27	2011	Case Report	Hypophosphatasia	Radiological, *in vitro* assays	*in vivo/vitro*	Homo sapiens	ALPL	SNP	n.a.	
28	2003	Cohort study	Bone marrow edema syndrome	Radiological, Histology, Serum	*in vivo*	Homo sapiens	osteocalcin<br>bone ALP<br>C-terminal cross-linking telopeptide<br>PIIANP	increased<br>increased<br>increased<br>increased	n.a.	mean bone-to-serum ratio
29	2017	Case series	Bone marrow edema syndrome	Radiological, Serum	*in vivo*	Homo sapiens	25(OH)D	decreased	n.a.	

30	2002	Case series	Bone marrow edema syndrome	Radiological, Serum	*in vivo*	Homo sapiens	lipoprotein (a)	decreased	n.a.	
							ApoA1	increased		
							ApoB	increased		
31	2000	Case series	Bone marrow edema syndrome	Serum	*in vivo*	Homo sapiens	lipoprotein (a)	increased	n.a.	
32	2007	Case report	Regional transient osteoporosis	Radiological	*in vivo*	Homo sapiens	vitamin C	decreased	n.a.	no reduced perfusion
33	2019	Cross-Sectional study	Bone marrow edema syndrome	Radiological, Serum	*in vivo*	Homo sapiens	bone-alkaline phosphatase (BAP)	increased	n.a.	decreased bone mineral density
							type I procollagen N-terminal propeptide (PINP)	increased		
							cross-linked N-telopeptide of type I collagen (NTx)	increased		
							tartrateresistant acid phosphatase-5b (TRACP-5b)	increased		
							pentosidine	increased		
34	2013	Case series	Bone marrow edema syndrome	Radiological, Serum	*in vivo*	Homo sapiens	lipoprotein (a)	increased	iloprost	
							ApoA1	Increased		
							ApoB	increased		
35	2005	Case series	hydroxyapatite deposition disease	Radiological	*in vivo*	Homo sapiens	n.a.	n.a.	n.a.	underlying the enthesis

n.a.: not applicable

References

1. Uetani, M.; Hashmi, R.; Ito, M.; Okimoto, T.; Kawahara, Y.; Hayashi, K.; Enomoto, H.; Shindo, H. Subchondral insufficiency fracture of the femoral head: magnetic resonance imaging findings correlated with micro-computed tomography and histopathology. J. Comput. Assist. Tomogr. 2003, 27, 189–193.
2. Matheny, J.B.; Goff, M.G.; Pownder, S.L.; Koff, M.F.; Hayashi, K.; Yang, X.; Bostrom, M.P.G.; van der Meulen, M.C.H.; Hernandez, C.J. An in vivo model of a mechanically-induced bone marrow lesion. J. Biomech. 2017, 64, 258–261.
3. Song, Y.; Cui, Y.; Zhang, X.; Lin, H.; Zhang, G.; Zeng, H.; Zeng, Y. Increased serum levels of MIC1/GDF15 correlated with bone erosion in spondyloarthritis: A pilot study. Medicine (Baltimore). 2018, 97, e13733.
4. Zhao, Z.; Wang, G.; Wang, Y.; Yang, J.; Wang, Y.; Zhu, J.; Huang, F. Correlation between magnetic resonance imaging (MRI) findings and the new bone formation factor Dkk-1 in patients with spondyloarthritis. Clin. Rheumatol. 2019, 38, 465–475.
5. Papuga, M.O.; Kwok, E.; You, Z.; Rubery, P.T.; Dougherty, P.E.; Pryhuber, G.; Beck, C.A.; Hilton, M.J.; Awad, H.A.; Schwarz, E.M. TNF is required for the induction but not the maintenance of compression-induced BME signals in murine tail vertebrae: limitations of anti-TNF therapy for degenerative disc disease. J. Orthop. Res. 2011, 29, 1367–1374.
6. Papuga, M.O.; Proulx, S.T.; Kwok, E.; You, Z.; Rubery, P.T.; Dougherty, P.E.; Hilton, M.J.; Awad, H.A.; Schwarz, E.M. Chronic axial compression of the mouse tail segment induces MRI bone marrow edema changes that correlate with increased marrow vasculature and cellularity. J. Orthop. Res. 2010, 28, 1220–1228.
7. Castillo-Gallego, C.; Aydin, S.Z.; Emery, P.; McGonagle, D.G.; Marzo-Ortega, H. Magnetic resonance imaging assessment of axial psoriatic arthritis: extent of disease relates to HLA-B27. Arthritis Rheum. 2013, 65, 2274–2278.

8. Appel, H.; Loddenkemper, C.; Grozdanovic, Z.; Ebhardt, H.; Dreimann, M.; Hempfing, A.; Stein, H.; Metz-Stavenhagen, P.; Rudwaleit, M.; Sieper, J. Correlation of histopathological findings and magnetic resonance imaging in the spine of patients with ankylosing spondylitis. Arthritis Res. Ther. 2006, 8, R143.
9. Soliman, E.; Labib, W.; el-Tantawi, G.; Hamimy, A.; Alhadidy, A.; Aldawoudy, A. Role of matrix metalloproteinase-3 (MMP-3) and magnetic resonance imaging of sacroiliitis in assessing disease activity in ankylosing spondylitis. Rheumatol. Int. 2012, 32, 1711–1720.
10. Issa, S.F.; Duer, A.; Ostergaard, M.; Horslev-Petersen, K.; Hetland, M.L.; Hansen, M.S.; Junker, K.; Lindegaard, H.M.; Moller, J.M.; Junker, P. Increased galectin-3 may serve as a serologic signature of pre-rheumatoid arthritis while markers of synovitis and cartilage do not differ between early undifferentiated arthritis subsets. Arthritis Res. Ther. 2017, 19, 80.
11. Boeters, D.M.; Nieuwenhuis, W.P.; Verheul, M.K.; Newsum, E.C.; Reijnierse, M.; Toes, R.E.M.; Trouw, L.A.; van der Helm-van Mil, A.H.M. MRI-detected osteitis is not associated with the presence or level of ACPA alone, but with the combined presence of ACPA and RF. Arthritis Res. Ther. 2016, 18, 179.
12. Waterborg, C.E.J.; Koenders, M.I.; van Lent, P.L.E.M.; van der Kraan, P.M.; van de Loo, F.A.J. Tyro3/Axl/Mertk-deficient mice develop bone marrow edema which is an early pathological marker in rheumatoid arthritis. PLoS One 2018, 13, e0205902.
13. Aaron, R.K.; Dyke, J.P.; Ciombor, D.M.; Ballon, D.; Lee, J.; Jung, E.; Tung, G.A. Perfusion abnormalities in subchondral bone associated with marrow edema, osteoarthritis, and avascular necrosis. Ann. N. Y. Acad. Sci. 2007, 1117, 124–137.
14. Libicher, M.; Ivancic, M.; Hoffmann, M.; Wenz, W. Early changes in experimental osteoarthritis using the Pond-Nuki dog model: technical procedure and initial results of in vivo MR imaging. Eur. Radiol. 2005, 15, 390–394.
15. Taljanovic, M.S.; Graham, A.R.; Benjamin, J.B.; Gmitro, A.F.; Krupinski, E.A.; Schwartz, S.A.; Hunter, T.B.; Resnick, D.L. Bone marrow edema pattern in advanced hip osteoarthritis: quantitative assessment with magnetic resonance imaging and correlation with clinical examination, radiographic findings, and histopathology. Skeletal Radiol. 2008, 37, 423–431.
16. Han, W.; Aitken, D.; Zheng, S.; Wang, B.; Wluka, A.E.; Zhu, Z.; Blizzard, L.; Wang, X.; Winzenberg, T.; Cicuttini, F.; et al. Higher Serum Levels of Resistin Are Associated With Knee Synovitis and Structural Abnormalities in Patients With Symptomatic Knee Osteoarthritis. J. Am. Med. Dir. Assoc. 2019.
17. Muratovic, D.; Findlay, D.M.; Cicuttini, F.M.; Wluka, A.E.; Lee, Y.R.; Edwards, S.; Kuliwaba, J.S. Bone marrow lesions in knee osteoarthritis: regional differences in tibial subchondral bone microstructure and their association with cartilage degeneration. Osteoarthr. Cartil. 2019.
18. Shabestari, M.; Vik, J.; Reseland, J.E.; Eriksen, E.F. Bone marrow lesions in hip osteoarthritis are characterized by increased bone turnover and enhanced angiogenesis. Osteoarthr. Cartil. 2016, 24, 1745–1752.
19. Wang, F.; Luo, A.; Xuan, W.; Qi, L.; Wu, Q.; Gan, K.; Zhang, Q.; Zhang, M.; Tan, W. The Bone Marrow Edema Links to an Osteoclastic Environment and Precedes Synovitis During the Development of Collagen Induced Arthritis. Front. Immunol. 2019, 10, 884.
20. Perry, T.A.; Parkes, M.J.; Hodgson, R.; Felson, D.T.; O'Neill, T.W.; Arden, N.K. Effect of Vitamin D supplementation on synovial tissue volume and subchondral bone marrow lesion volume in symptomatic knee osteoarthritis. BMC Musculoskelet. Disord. 2019, 20, 76.
21. Kwoh, C.K.; Roemer, F.W.; Hannon, M.J.; Moore, C.E.; Jakicic, J.M.; Guermazi, A.; Green, S.M.; Evans, R.W.; Boudreau, R. Effect of oral glucosamine on joint structure in individuals with chronic knee pain: a randomized, placebo-controlled clinical trial. Arthritis Rheumatol. (Hoboken, N.J.) 2014, 66, 930–939.
22. Shinmura, K.; Ishida, T.; Goto, T.; Kuroda, M.; Hattori, H.; Nagai, S.; Imamura, T.; Mukai, K.; Imakiire, A. Expression of cyclooxygenase-2 in chondroblastoma: immunohistochemical analysis with special emphasis on local inflammatory reaction. Virchows Arch. 2004, 444, 28–35.
23. Wen, Q.; Ma, L.; Chen, Y.-P.; Yang, L.; Luo, W.; Wang, X.-N. A rabbit model of hormone-induced early avascular necrosis of the femoral head. Biomed. Environ. Sci. 2008, 21, 398–403.
24. Wu, R.-W.; Wang, F.-S.; Ko, J.-Y.; Wang, C.-J.; Wu, S.-L. Comparative serum proteome expression of osteonecrosis of the femoral head in adults. Bone 2008, 43, 561–6.
25. Bosco, G.; Vezzani, G.; Mrakic Sposta, S.; Rizzato, A.; Enten, G.; Abou-Samra, A.; Malacrida, S.; Quartesan, S.; Vezzoli, A.; Camporesi, E. Hyperbaric oxygen therapy ameliorates osteonecrosis in patients by modulating inflammation and oxidative stress. J. Enzyme Inhib. Med. Chem. 2018, 33, 1501–1505.

26. Whyte, M.P.; Wenkert, D.; McAlister, W.H.; Mughal, M.Z.; Freemont, A.J.; Whitehouse, R.; Baildam, E.M.; Coburn, S.P.; Ryan, L.M.; Mumm, S. Chronic recurrent multifocal osteomyelitis mimicked in childhood hypophosphatasia. J. Bone Miner. Res. 2009, 24, 1493–1505.
27. Mentrup, B.; Marschall, C.; Barvencik, F.; Amling, M.; Plendl, H.; Jakob, F.; Beck, C. Functional characterization of a novel mutation localized in the start codon of the tissue-nonspecific alkaline phosphatase gene. Bone 2011, 48, 1401–1408.
28. Berger, C.E.; Kroner, A.H.; Minai-Pour, M.B.; Ogris, E.; Engel, A. Biochemical markers of bone metabolism in bone marrow edema syndrome of the hip. Bone 2003, 33, 346–351.
29. Horas, K.; Fraissler, L.; Maier, G.; Jakob, F.; Seefried, L.; Konrads, C.; Rudert, M.; Walcher, M. High Prevalence of Vitamin D Deficiency in Patients With Bone Marrow Edema Syndrome of the Foot and Ankle. Foot ankle Int. 2017, 38, 760–766.
30. Berger, C.E.; Kroner, A.; Stiegler, H.; Erdel, M.; Haas, O.A.; Engel, A. Hypofibrinolysis, lipoprotein(a), and plasminogen activator inhibitor. Clin. Orthop. Relat. Res. 2002, 342–349.
31. Berger, C.E.; Kluger, R.; Urban, M.; Kowalski, J.; Haas, O.A.; Engel, A. Elevated levels of lipoprotein(a) in familial bone marrow edema syndrome of the hip. Clin. Orthop. Relat. Res. 2000, 126–131.
32. Rodriguez, S.; Paniagua, O.; Nugent, K.M.; Phy, M.P. Regional transient osteoporosis of the foot and vitamin C deficiency. Clin. Rheumatol. 2007, 26, 976–978.
33. Ota, S.; Chiba, D.; Sasaki, E.; Kumagai, G.; Yamamoto, Y.; Nakaji, S.; Tsuda, E.; Ishibashi, Y. Symptomatic bone marrow lesions induced by reduced bone mineral density in middle-aged women: a cross-sectional Japanese population study. Arthritis Res. Ther. 2019, 21, 113.
34. Anagnostakos, K.; Orth, P. Role of serum lipoprotein at the site of iloprost therapy in the treatment of painful bone marrow edema. Orthopedics 2013, 36, e1283-9.
35. Bui-Mansfield, L.T.; Moak, M. Magnetic resonance appearance of bone marrow edema associated with hydroxyapatite deposition disease without cortical erosion. J. Comput. Assist. Tomogr. 2005, 29, 103–107.
